# Acute Viral Gastrointestinal (GI) Infections in the Tropics—A Role for Cartridge-Based Multiplex PCR Panels?

**DOI:** 10.3390/tropicalmed7050080

**Published:** 2022-05-19

**Authors:** Stefanie Kramme, Theo Dähne, Alexey Fomenko, Marcus Panning

**Affiliations:** 1 Institute for Infection Prevention and Hospital Epidemiology, University Medical Center Freiburg, Faculty of Medicine, University of Freiburg, 79106 Freiburg, Germany; stefanie.kramme@uniklinik-freiburg.de; 2 Institute of Virology, University Medical Center Freiburg, Faculty of Medicine, University of Freiburg, 79104 Freiburg, Germany; theo.daehne@uniklinik-freiburg.de (T.D.); alexey.fomenko@uniklinik-freiburg.de (A.F.)

**Keywords:** molecular methods, multiplex syndromic panels, acute viral gastroenteritis, resource-poor settings, tropics

## Abstract

Acute gastroenteritis (AGE) contributes to increased morbidity and mortality worldwide. In particular, children in resource-poor settings suffer from frequent episodes of diarrhea. A variety of pathogens, including bacteria, viruses, fungi, and protozoa, can cause AGE. Common viruses associated with AGE are norovirus, rotavirus, astrovirus, adenovirus, and sapovirus. Due to their similar clinical presentation, AGE pathogens cannot be distinguished on clinical grounds rendering the etiological diagnosis challenging. However, reliable diagnosis is essential for individual and public health reasons, e.g., to limit transmission, for appropriate antibiotic use, prognostic appreciation, and vaccination programs. Therefore, high-quality data derived by accurate diagnostics are important to improve global health. In Western industrialized countries, diagnosis relies on microbiological testing, including culture methods, microscopy, immunochromatography, and single-target molecular methods. Recently, multiplex PCR or syndromic panels have been introduced, which simultaneously analyze for multiple pathogens in a very short time. A further technological advancement is cartridge-based syndromic panels, which allow for near patient/point-of-care testing independently from a laboratory. In resource-poor tropical regions, however, laboratory diagnosis is rarely established, and there are little routine laboratory data on the epidemiology of viral AGE pathogens. Limiting factors for the implementation of syndromic panels are high costs, sophisticated equipment, and the need for trained personnel. In addition, pilot studies have shown a large number of viral (co-)detections among healthy controls, thus further challenging their clinical utilization. Hence, there are little evidence-based data on the impact of multiplex syndromic panels from resource-limited regions. Here, we aim to provide a brief overview of what is known about the use of syndromic panels for virus-associated AGE in tropical regions and to address future challenges.

## 1. Introduction

Acute gastroenteritis (AGE) is associated with a high disease burden worldwide. On a global scale, it is estimated that 2 billion cases occur each year [1].

In industrialized countries, AGE has a great impact on individual and public health but to a lesser extent on mortality. Here, the elderly population and immunosuppressed patients are at the highest risk for severe and sometimes fatal courses [2,3,4]. A wide range of pathogens, including bacteria, viruses, fungi, and protozoa, can cause AGE.

On the contrary, in resource-limited regions, such as low- and middle-income countries (LMIC), morbidity and mortality can be very high and account for a considerable proportion of emergency department visits, hospitalizations, and, ultimately, deaths. Children, in particular, constitute the most vulnerable population in tropical regions; around 2 million deaths occur each year, mainly those with malnutrition [5].

Based on data from a large multicentric children cohort study from eight resource-poor sites (Bangladesh, Brazil, India, Nepal, Pakistan, Peru, South Africa, and Tanzania), a predominant viral cause (36.4%) could be identified by quantitative RT-PCR, followed by bacterial (25.0%) and parasitic (3.5%) (Etiology, Risk Factors, and Interactions of Enteric Infections and Malnutrition and the Consequences for Child Health and Development (MAL-ED), [6]).

Common viruses associated with childhood AGE in the resource-poor settings, in descending order, are rotavirus, enteric adenovirus (types 40/41), sapovirus, norovirus, and astrovirus [6,7,8].

Virus transmission is facilitated by unfavorable socioeconomic levels with cramped and crowded households, malnutrition, contaminated water or food, poor sanitation and hygiene, and climatic conditions that allow environmental contamination by pathogens [9]. All of these could possibly explain the high disease burden of AGE in developing countries, where large and explosive outbreaks can occur (e.g., in refugee camps) [10]. Outbreaks can also occur, e.g., in hospitals, long-term care facilities (LTCF), or nursing homes.

Traditional methods for the detection of AGE pathogens include laborious and time-consuming stool cultures, antigen detection methods, and microscopy. In addition, all of these traditional techniques are less sensitive and specific compared to nucleic acid amplification techniques (NAT) [11]. However, timely diagnosis may prevent adverse patient outcomes and stop further spread by rapid isolation precautions, e.g., in a hospital or an LTCF setting. Patients may benefit from rapid and appropriate diagnosis, avoiding erroneous prescription of antibiotics, concomitant side effects, and antibiotic-resistance related issues. Of note, a study with 414 patients performed in India using a molecular multiplex panel registered the use of antibiotics in 90% of patients with a viral causative agent [12].

Molecular methods have undoubtedly revolutionized clinical virology in the past decades and helped to improve our understanding of the epidemiology of virus-associated AGE. Using NAT, norovirus was shown to be a key AGE pathogen causing nearly a fifth of all cases of AGE [13]. With the advent of recently introduced multiplex PCR panels, a comprehensive and simultaneous analysis of up to 20 different pathogens is possible within a very short time and minimal hands-on time. This allows for a syndromic testing approach since these panels test for pathogens associated with a specific infectious syndrome. As of yet, these panels are primarily available for respiratory tract and gastrointestinal infections. Although multiplex testing is increasingly utilized in many industrialized countries, data from, e.g., Africa or tropical settings, are scarce.

Data on the clinical impact (i.e., antibiotic prescription, auxiliary testing, isolation, length of hospital stay) of syndromic panels, even from industrialized regions, are lacking at large and are, to some extent, controversial. According to the health technology assessment report by Freeman et al., multiplex testing for gastrointestinal pathogens leads to a higher number of positive results than conventional testing. However, the clinical significance of these additional positive results is unclear as test-treatment trials are lacking. Therefore, no conclusion can be drawn whether the additional positive results lead to overdiagnosis or improved patient management [14].

Further important hurdles for their implementation are high costs, the need for sophisticated equipment, and the lack of evidence-based criteria for their clinical use. Especially for bacterial AGE pathogens, it is imperative to discriminate between true infection and colonization. Quantitative data from PCR-based methods might help to distinguish active infection from colonization but warrant careful interpretation and standardization [6,15,16]. Unlike bacterial AGE, there is no approved therapy for most viral infections, except for symptomatic treatment and isolation. Importantly, rotavirus vaccines are now widely used for the prevention of rotavirus-associated gastroenteritis. To control the success of vaccination programs, reliable detection methods are of importance. This gives rise to the notion that molecular methods are not only important for individuals but also for public health in many countries worldwide. Finally, the cost-effectiveness of molecular methods is hard to assess not only in resource-rich countries, as prerequisites as laboratory equipment and test costs, reimbursement, and other variables are significantly different from country to country. Having this in mind, it is evident that clinical guidelines do not recommend universal testing of all patients presenting with diarrheal symptoms.

Here, we summarize what is known about the use of multiplex syndromic panels for the detection of virus-associated AGE in resource-limited regions.

## 2. Methods

We conducted a literature search using the terms shown in Table 1. We searched the Medline database and retrieved a total of 596 studies. We included 27 articles and our own experience to set up a brief narrative review on the use of syndromic panels for virus-associated AGE in tropical regions [5,6,12,15,16,17,18,19,20,21,22,23,24,25,26,27,28,29,30,31,32,33,34,35,36]. Exclusion criteria were population outside LMIC and singleplex PCR; we did not apply any year filters or age restrictions to the target population. We focused on technical aspects and the clinical impact of syndromic panels. Finally, we suggest directions for future research.

## 3. Technical and Pre-Analytical Considerations

In general, before the introduction of novel diagnostic methods in a laboratory or hospital, it is advisable to perform change management to define, e.g., the clinical needs, the indication to order a test, and to anticipate possible pitfalls [37]. Loderstadt and colleagues present a detailed overview of various issues, including regulatory ones for the molecular detection of bacteria associated with AGE in the tropics [38].

The challenges in AGE diagnosis are partly explainable by the fact that stool is a complex material due to its variable content of PCR inhibitors, which depend on the patient’s diet, gut microbiome, lifestyle, and environmental factors such as exposure to animals or unimproved sanitation infrastructure [38,39]. Substances such as urea, hemoglobin, heparin, polysaccharides, chlorophyll from vegetables, bile salts, and glycolipids have been identified as inhibitors and are possible factors for false-negative results [39,40]. Especially in the tropical setting, this challenge is considerable since here, necessary requirements such as refrigerated or frozen storage and transport are not always guaranteed, either due to a lack of logistics or interruptions in the cold chain due to electrical interruptions, which poses the risk of degradation of the unextracted nucleic acid in the samples due to suboptimal storage conditions. Attempts have been made to counteract this problem through sample storage on Flinders Technology Associates^®^ (FTA) cards at ambient temperature. The FTA^®^ paper card is a fiber-membrane-based system impregnated with chemicals that lyse cells, cell nuclei, organelles, and nucleic acid. The released nucleic acids bind tightly in the fibers of the FTA^®^ matrix, which protects the nucleic acids from damaging substances such as nucleases and thus reduces degradation [41]. The application of this technology for storage has been evaluated primarily for bacterial DNA in stool samples, as viral RNA is considered an unstable molecule due to the ubiquitous presence of RNAases. Hence, a systematic review of FTA^®^ cards for the preservation of viral RNA found only one trial in which FTA^®^ cards were used for stool samples. Here, promising results were shown for rotavirus detection both in vitro under different storage temperatures and under field conditions, while another study published after the search of the systematic review investigated different enteropathogens [22,42]. The study showed that FTA^®^ card-based storage has the lowest sensitivity for noroviruses compared to bacterial pathogens [43]. A promising, cost-effective alternative to commercially available paper matrix cards for the storage and preservation of stool specimens was proposed by Cromeans et al., who modified universal nucleic acid extraction buffer (UNEX) to impregnate a self-developed cellulose filter disc (UNEXP disc). Suspensions of norovirus-positive stool samples (n = 54) were applied to UNEXP discs; after two weeks of storage at room temperature on the UNEXP discs, all samples showed norovirus RT-qPCR positive. A subset of seven samples was stored for three months and found RT-qPCR positive. In addition, genotyping was successful in 76% (41/54) of the samples [44]. Further research on FTA^®^ card and UNEXP cards for other AGE viruses and under different storage conditions and durations would therefore be desirable.

Technically, a wide variety of molecular laboratory-developed tests (LDT) as well as commercial assays are available to detect AGE pathogens [45]. The Luminex GPP assay was one of the first syndromic assays for GI pathogens on the market. It is a truly laboratory-based workflow, as it requires separate nucleic acid extraction and (in previous versions) post-PCR processing steps. In a similar fashion, the Fast Track Diagnostics gastroenteritis panel offers real-time PCR-based multiplex testing. These conventional multiplex panels allow for batch testing of up to 96 samples but require sophisticated technical equipment. These technical prerequisites obviously limit their application in remote and/or resource-limited regions. However, a recent study showed that the detection rate increased over conventional methods [12]. A real-time PCR system is not available in many tropical regions due to cost, lack of equipment, and high technical demand on the staff, and therefore, the data on the routine use of PCR for AGE diagnosis are limited [25].

Most studies evaluating AGEs in resource-limited settings with multiplex PCR transfer the stool samples to industrialized countries or occasionally to remote urban laboratories with sufficient infrastructure [30,36,46].

The majority of studies are epidemiological and address the question of detection of coinfection in AGE in specific regions.

However, the question of the utility of multiplex diagnostics in the LMIC setting is unclear, and in particular, the frequent multiple pathogen detection complicates this circumstance by making treatment decisions difficult. It is conceivable that the possibility of differentiating between bacterial and viral pathogens may reduce the use of antibiotics. However, if multiple pathogens are detected without differentiation between infection and colonization, the opposite is also conceivable with increased costs due to antibiotic overuse and the development of antimicrobial resistance [29].

Likewise, the question of cost-effectiveness analysis of conventional multiplex PCR diagnostics for the detection of AGE in resource-limited settings is largely unanswered. For example, only a study conducted in India by Mitra et al. showed that similar test accuracy can be achieved at a lower cost using in-house designed multiplex PCR compared to commercially available real-time assay (FTD Viral Gastroenteritis kit) [35].

Technological advancements are cartridge-based syndromic panels, i.e., no separate nucleic acid extraction and amplification/detection steps are necessary. This approach allows for rapid and point of care testing independently from diagnostic laboratories. These panels display very fast turn-around and minimal hands-on time but require specialized equipment from the respective manufacturer [47]. Currently, the BioFire Film Array Gastrointestinal Panel and the QIAstat Gastrointestinal (GI) Panel are examples of commercially available cartridge-based assays (Table 2).

Pre-analytical considerations should also include the panel composition and the epidemiology of the analyzed pathogens. Major viral targets included in AGE multiplex panels are adenovirus 40/41, astrovirus, norovirus genogroup I/II, rotavirus A, and, in some assays, sapovirus. These pathogens represent the most important viral causes of AGE (Table 3). Before the introduction of a novel diagnostic technique, the analytical sensitivity or the limit of detection needs to be known for every target. Comparison studies of current syndromic panels have demonstrated equivalent or even superior performance to reference assays [45,48,49]. Not surprisingly, performance data varied between panels but also within panels with respect to specific pathogens [50,51]. Frequently, overall sensitivity is shown but might be critically low for selected targets [17]. In addition, genotypes can influence performance. For adenovirus, the most common types seen in AGE are included (types 40/41) in most assays. However, regionally circulating virus types might influence the reliability of results. Critically, validation studies conducted in tropical regions remain scarce but are of importance. Commercial panels include fixed combinations of pathogens and do not allow them to be customized, e.g., to local needs or seasonality of pathogens. It is well known that in temperate regions, norovirus and rotavirus typically occur in the colder month of the year (“winter vomiting disease”). In tropical regions, however, explosive outbreaks can occur with hundreds of cases due to the consumption of contaminated water irrespective of seasonality. On the contrary, selected pathogens may be rare overall, and consequently, the positive predictive value is very low. In addition, the introduction of the rotavirus vaccine also has an impact on the pretest probability and should be taken into account. Thus, knowledge about patients‘ vaccination status is of interest since rotavirus vaccination may also yield a positive PCR result for an indeterminate time period post vaccination [52]. In this respect, LDTs allow for greater flexibility as only locally relevant pathogens can be included. Finally, many available assays do have a CE label, which limits, e.g., the selection of the sample specimen type to those endorsed by the manufacturer. If alternative specimens are to be tested, extensive validation needs to be done to account for regulatory issues.

One of the most promising benefits of the new syndromic cartridge-based panels is the reduced hands-on and turn-around time. They can provide comprehensive results in <2 h. This might lead to rapid and more targeted therapy, more informed decision making regarding isolation, and optimized patient management [53].

## 4. Clinical Impact and Current Evidence

As with any diagnostic testing, the indication should be inferred in light of local epidemiological considerations and the clinical presentation. Current reviews do not universally recommend diagnostic multiplex testing in every patient with suspected diarrheal diseases [54]. Our literature search yielded only 13 studies reporting on the use of multiplex PCR panels for AGE in tropical settings. Critically, many of these studies were conducted in tropical regions, but the multiplex analysis was performed by study partners, e.g., in Europe [15,17]. In addition, these studies were not designed to assess the clinical impact of multiplex testing on patient care and public health. This certainly limits the interpretation of the usability of multiplex testing in tropical settings.

In industrialized regions, a study evaluated the impact of another platform, the FilmArray GIP, on length of stay and patient isolation. This study found that patients spent considerable time without appropriate isolation methods in the hospital, whereas other patients were placed under contact precautions without cause [53]. Another study has shown that costs for the clinical laboratory increased, but overall, there were savings for the hospital [55]. Claas et al. reported that 65% of positive results were from pathogens not specifically ordered by the treating physician [56]. The clinical impact is much harder to assess in resource-limited settings where it became apparent that in children, numerous pathogens can be detected in cases with diarrhea as well as in asymptomatic controls [17]. Another study on infectious diarrhea including adult patients with persistent diarrhea and asymptomatic controls came to a similar conclusion in that positive results need confirmation of an active infection state [5]. These findings emphasize that it requires careful analysis of the local setting (i.e., patient population: adult versus pediatric, ambulatory versus in-hospital, immune status/previous antibiotic treatment, travel associated) and to evaluate the implementation of new methods in the local laboratory. The integrated stewardship concept addresses these concerns and might help in decision making when implementing syndromic panels [57].

For some patients, a more tailored approach would be more effective but not possible with the fixed combinations delivered by the manufacturers. It seems advisable to develop algorithms, which patient should be tested using which method. This apparently needs a cross-sectional and collaborative effort of all involved partners within a hospital or in primary care. However, in industrialized settings and even more so in resource-limited settings, there are still limited evidence-based data available to support a favorable benefit–risk ratio. In a meta-analysis including 162 norovirus outbreaks, implementation of infection control measures reduced patient case counts and length of hospital stay 0.6 and 0.7 fold, respectively, emphasizing the value of rapid and accurate diagnostics [58]. However, these data lack the economic impact, which is of particular relevance for low-income settings.

To conclude, although syndromic panels have been available for some time, there are still no general recommendations for the routine use of multiplex syndromic panels in patients presenting with AGE neither in industrialized nor in resource-poor settings. Thus, the question of the role of syndromic panels in the tropics remains open.

## 5. Outlook and Perspectives

Syndromic panels represent the spearhead of novel diagnostic tools not only for AGE. They have been proven to deliver fast and accurate results in multiple comparison studies and will help to advance our understanding of the epidemiology of AGE. This might lead to improved patient care but needs further (ideally multicenter and prospective) studies in, e.g., different settings, specific patient groups, and geographical locations. High costs of syndromic panels have significantly hindered their widespread implementation in routine diagnostic laboratories. This holds even more true for their use in resource-poor countries. Therefore, cost-effectiveness analyses are needed to allow state-of-the-art testing in remote settings. In addition, future research should be dedicated to coming to evidence-based recommendations on their use in different settings. Finally, cost-effectiveness and shortcomings have to be communicated between the laboratory, clinicians, practitioners, and other stakeholders.

## Figures and Tables

**Table 1:** Literature search strategy for MEDLINE.

Search No	Search Strategy
1	africa* [Title/Abstract]
2	resource-poor [Title/Abstract]
3	tropic* [Title/Abstract]
4	(low income [Title/Abstract]) OR (middle income [Title/Abstract])
5	equatorial region [Title/Abstract]
6	developing countries [Title/Abstract]
7	“Poverty Areas” [Mesh]
8	“Africa” [Mesh]
9	“Developing Countries” [Mesh]
10	“Molecular Diagnostic Techniques” [Mesh]
11	“Polymerase Chain Reaction” [Mesh]
12	“Real-Time Polymerase Chain Reaction” [Mesh]
13	“Reverse Transcriptase Polymerase Chain Reaction” [Mesh]
14	“Multiplex Polymerase Chain Reaction” [Mesh]
15	“Nucleic Acid Amplification Techniques” [Mesh]
16	PCR [Title/Abstract] OR polymerase chain reaction [Title/Abstract] OR nucleic acid amplification [Title/Abstract]
17	(syndromic panel* [Title/Abstract]) OR (syndromic multiplex [Title/Abstract]) OR (syndromic test* [Title/Abstract])
18	(cartridge-based real-time PCR[Title/Abstract]) OR (cartridge-based PCR[Title/Abstract]) OR (cartridge-based test* [Title/Abstract]) OR (cartridge-based NAAT [Title/Abstract]) OR (cartridge-based Nucleic Acid Amplification Test [Title/Abstract])
19	“Viruses” [Mesh]
20	viral [Title/Abstract] OR virus [Title/Abstract]
21	“Sapovirus” [Mesh]
22	“Rotavirus Infections” [Mesh]
23	“Rotavirus” [Mesh]
24	“norovirus” [MeSH Terms]
25	“Avastrovirus” [Mesh]
26	“Adenoviridae” [Mesh]
27	“Adenoviridae Infections” [Mesh]
28	“Astroviridae” [Mesh]
29	sapovir* or rotavir* or norovir* or astrovir* or adenovir*
30	“Gastroenteritis” [Mesh]
31	“Diarrhea” [Mesh]
32	gastroenteritis [Title/Abstract] OR enteritis [Title/Abstract] OR gastrointestinal infec-tion [Title/Abstract] OR diarrhea [Title/Abstract] OR travellers disease [Title/Abstract]
33	#1 OR #2 OR #3 OR #4 OR #5 OR #6 OR #7 OR #8 OR #9
34	#10 OR #11 OR #12 OR #13 OR #14 OR #15 OR #16 OR #17 OR #18
35	#19 or #20 or #21 or #22 or #23 or #24 or #25 or #26 or #27 or #28 or #29
36	#30 or #31 or #32
37	#33 and #34 and #35 and #36

**Table 2:** Currently available cartridge-based commercial multiplex panels for AG pathogens.

Manufacturer	Gastrointestinal Panel
Biomerieux	FilmArray Gastrointestinal (GI) Panel
Luminex	Verigene Enteric Pathogens Test
Qiagen	QIAstat-Dx Gastrointestinal Panel

**Table 3:** Baseline characteristics of frequently included pathogens in multiplex PCR panels.

Virus	Mode of Transmission	Target Population	Incubation Period (Days)	Clinical Significance	Vaccination
Sapovirus	Fecal–oral; food and water	Infants and young children; adults less frequently	1–2	Mild to moderate gastroenteritis	No
Rotavirus	Fecal–oral; food and water	Infants and young children; adults less frequently	1–2	Moderate to severe gastroenteritis, including severe dehydration; hospital-acquired infections occur	Yes
Norovirus	Fecal–oral; food and water; possibly via aerosols	All age groups	1–2	Moderate to severe gastroenteritis, including hospital-acquired infections; prolonged shedding in immunocompromised patients	No
Astrovirus	Fecal–oral; food and water	Young children; adults less frequently	1–2	Mild to moderate gastroenteritis, including asymptomatic infections	No
Adenovirus	Fecal–oral; food and water	Infants and young children; adults less frequently	>7	Moderate to severe gastroenteritis, including hospital-acquired infections; possible persistence in immunocompromised patients	No

## Data Availability

Not applicable.
